# Physical Characteristics Pertaining to Health-Related Quality of Life in Hospitalized Patients With Hematologic Malignancies: A Cross-Sectional Study

**DOI:** 10.7759/cureus.91063

**Published:** 2025-08-26

**Authors:** Hiroki Kofuji, Tomoki Aoyama, Masakatsu Hishizawa, Hiroko Miyazaki

**Affiliations:** 1 Department of Human Health Sciences, Graduate School of Medicine, Kyoto University, Kyoto, JPN; 2 Department of Rehabilitation Medicine, Kyoto Katsura Hospital, Kyoto, JPN; 3 Department of Hematology, Kyoto Katsura Hospital, Kyoto, JPN

**Keywords:** body composition, cancer patients, gait speed, health-related quality of life (hrqol), hematologic malignancies (hm), physical function

## Abstract

Background

Patients with hematologic malignancies often experience pre-existing physical decline, which is further exacerbated during chemotherapy, resulting in markedly reduced health-related quality of life (HRQOL). These functional impairments are largely attributable to modifiable factors such as physical inactivity and malnutrition. However, the precise relationships among body composition, physical function, and HRQOL in this population remain insufficiently understood. Therefore, this study aimed to identify key physical determinants of HRQOL in hospitalized patients with hematologic malignancies.

Methods

A total of 62 patients with hematologic malignancies who were hospitalized for chemotherapy were included in the analysis. Partial correlation analyses were conducted to examine the associations between physical characteristics and HRQOL, assessed using the health state utility value (HSUV) from the EuroQol 5-Dimension 5-Level (EQ-5D-5L). Analyses were adjusted for age, sex, hemoglobin concentration, and steroid dosage. Physical parameters included body composition, grip strength, gait speed, gait independence, and standing balance. Additionally, multiple linear regression analysis was performed to identify independent predictors of HSUV. Variables with statistical or clinical relevance were included.

Results

The mean age of participants was 71.7 ± 13.3 years, and the mean HSUV was 0.799 ± 0.140. Partial correlation analysis showed significant associations between HSUV and body weight, BMI, body fat percentage, gait speed, and gait independence. Additionally, multiple linear regression analysis identified gait speed (β = 0.304, p = 0.047) and gait independence (β = 0.411, p = 0.005) as independent predictors, explaining 50.5% of the variance in HSUV.

Conclusion

Gait speed and independence are critical indicators of HRQOL in hospitalized patients with hematologic malignancies. Assessing these simple functional measures may help identify patients at risk of poor HRQOL, underscoring the importance of integrating physical rehabilitation into standard supportive care.

## Introduction

Hematologic malignancies are diseases for which a cure is often expected through chemotherapy, allowing many patients to achieve long-term survival [1,2]. Nonetheless, their global incidence has been steadily increasing over the past few decades [3]. Although survival has improved, hospitalized patients with hematologic malignancies continue to exhibit significantly lower muscle mass and physical function compared to healthy individuals, indicating persistent functional impairments [4]. Moreover, nearly half of patients with hematologic malignancies present with sarcopenia before treatment, which independently predicts poorer overall and progression-free survival [5]. In this context, exercise during chemotherapy for hematologic malignancies has received growing attention. A systematic review confirmed its safety and feasibility in hospitalized patients, and preliminary findings suggest possible benefits for physical function, fatigue, and health-related quality of life (HRQOL), including the potential to help maintain it during treatment [6].

In cancer treatment, it is recommended to consider objective indicators (such as survival rates) and subjective indicators (such as HRQOL) [7,8]. The importance of assessing patient health status using patient-reported outcome measures has also been highlighted [9]. One of the most fundamental components of HRQOL is the daily living function [10], which is expected to improve through medical interventions [11]. Therefore, enhancing the patients' functional capacity through physical therapy may lead to improvements in their HRQOL.

Previous studies have reported that the HRQOL of patients with hematologic malignancies is poorer than that of the general population. A systematic review concluded that multiple dimensions of HRQOL are significantly lower in these patients compared to healthy peers [12]. Similarly, a cross-sectional study found that HRQOL in onco-hematological patients was significantly worse than in the general population [13]. Moreover, recent research focusing on hospitalized patients revealed marked HRQOL impairments, with high levels of fatigue and appetite loss reported during inpatient care [14]. These findings underscore that, even while undergoing curative treatments, patients often experience a substantial symptom burden and reduced HRQOL.

Importantly, emerging evidence has identified several modifiable physical and nutritional factors associated with HRQOL in this patient group. For example, one study found that, compared to patients with higher hemoglobin levels, those with low levels reported greater fatigue and dyspnea, lower muscle strength, greater difficulty with daily activities, and worse overall QOL [15]. This suggests that anemia-related weakness can negatively impact functional status and HRQOL. Furthermore, highlighting the profound influence of physical inactivity, one study observed significant muscle wasting and weakness in hospitalized patients with hematologic malignancies, a decline attributed more to disuse than to cancer cachexia [4]. In other words, prolonged illness-related inactivity is a major contributor to muscle dysfunction. Consistent with this, low physical activity levels and poor nutritional status are recognized as key contributors to diminished muscle function in patients undergoing chemotherapy for hematologic malignancies [16].

These findings highlight that rehabilitation-relevant factors, such as maintaining physical activity and ensuring nutritional support, play a crucial role in determining a patient’s physical function. Encouragingly, evidence indicates that interventions targeting these modifiable factors may yield clinically meaningful benefits. A prospective study, for example, demonstrated the positive effects of high-frequency, low-intensity exercise during chemotherapy. Patients in this group not only maintained their muscle function but also showed improvements in physical performance, activities of daily living, and HRQOL, along with reduced psychological distress. In stark contrast, patients with lower exercise frequency experienced functional decline [17].

Taken together, these data indicate that timely rehabilitation targeting modifiable factors is essential for mitigating physical decline and improving HRQOL in patients with hematologic malignancies. From a clinical standpoint, interventions should prioritize factors that can be readily managed through rehabilitation, such as deconditioning and malnutrition. While anemia may be less amenable to such approaches, addressing these other modifiable elements remains a cornerstone of comprehensive supportive care.

Guidelines suggest that a combination of aerobic and moderate-intensity resistance training can effectively alleviate cancer-related fatigue (CRF), a common and debilitating symptom among hospitalized cancer patients [18]. The management of CRF plays a key role in improving patients' daily functioning and overall well-being. Furthermore, a recent systematic review and meta-analysis of randomized controlled trials in patients with hematologic malignancies concluded that exercise interventions significantly improve HRQOL, most notably by enhancing emotional functioning and alleviating pain [19]. Collectively, the evidence suggests that by alleviating fatigue and potentially improving appetite, maintaining higher physical function and fitness is associated with better HRQOL in patients with hematologic cancers. In other words, patients who maintain higher levels of physical strength and activity during treatment report better quality of life, likely due to reduced symptom burden and preserved functional independence.

Despite these findings, the complex interrelationships among body composition, physical function, and HRQOL in patients with hematologic malignancies remain insufficiently understood. In particular, it is unclear which physical and nutritional factors are both associated with HRQOL and modifiable through rehabilitation. Clarifying these associations could help develop effective, targeted interventions for improving patient outcomes. Therefore, this cross-sectional descriptive study aimed to examine the associations among clinical variables, body composition, physical function, and HRQOL in hospitalized patients with hematologic malignancies undergoing either initial or repeated cycles of chemotherapy. To achieve this, both correlation and multivariable regression analyses were used to identify key factors that could guide the development of targeted rehabilitation strategies.

## Materials and methods

Study design and participants

This cross-sectional observational study was conducted during inpatient chemotherapy and included both descriptive and analytical analyses. A total of 106 newly diagnosed patients with hematologic malignancies were consecutively recruited between March 2023 and October 2023 at Kyoto Katsura Hospital in Kyoto, Japan. The participants were diagnosed with hematologic malignancies, including malignant lymphoma, myelodysplastic syndrome, acute leukemia, multiple myeloma, and chronic leukemia. All assessments were conducted during the current hospitalization but prior to the initiation of the scheduled chemotherapy cycle. The study sample included both patients who were chemo-naive and those who had received chemotherapy before this admission. The exclusion criteria included bed rest restrictions, difficulty maintaining a standing position without support, cognitive impairment, inability to complete all assessments, and a positive COVID-19 test.

Assessments were conducted before chemotherapy initiation to exclude the effects of chemotherapy. Data collection was conducted by the first author, a physical therapist with 13 years of clinical experience. All assessments, including grip strength, gait speed, gait independence, and standing balance, were directly measured by the researcher, not extracted from medical records. To ensure patient safety, the evaluation was discontinued if the neutrophil count was less than 500/μL (due to a high risk of infection) or if the platelet count was less than 10,000/μL (due to a high risk of bleeding). This study adhered to the principles of the Declaration of Helsinki concerning research involving human subjects and was reviewed and approved by the Ethics Committee of the Graduate School of Medicine, Faculty of Medicine, Kyoto University Hospital, with permission obtained from the head of the institution (Approval No. R4593). The primary evaluation items were physical function and body composition, which were investigated using multiple control variables to assess their association with health state utility values (HSUV) from the EuroQol-5 Dimension 5-level (EQ-5D-5L) questionnaire.

Assessments

Demographic, Clinical, and Diagnostic Data

The following data were extracted from the medical records of each patient: age, sex, height, weight, body mass index (BMI), blood test results (albumin (Alb) and hemoglobin (Hb) levels), diagnosis of hematological diseases as the underlying condition, prior chemotherapy rounds, and steroid dosage. HRQOL, body composition, physical function, and cognitive function were assessed. BMI was measured using an automated height and weight scale (AD-6228AP; A&D Company Limited, Tokyo, Japan). Body weight and height were measured in the morning, typically before 10:00 AM. All patients wore standard hospital gowns, and 0.5 kg was subtracted from the measured weight to account for gown weight, in accordance with institutional protocol. These measurements were conducted on the same day as the physical function assessments, prior to the initiation of chemotherapy. Blood test results, including Alb and Hb levels, were obtained from routine peripheral blood tests conducted on the same day or within 24 hours prior to the evaluation of body composition, physical function, and HRQOL.

HRQOL

The quality of life (QOL) was assessed using the Japanese version of the EQ-5D-5L questionnaire [20,21]. Permission to use the EQ-5D-5L was obtained from the EuroQol Research Foundation. The questionnaire comprises five items: mobility, self-care, usual activities, pain/discomfort, and anxiety/depression. In the EQ-5D-5L, patients are asked to choose one of five levels for each item: no problems, slight problems, moderate problems, severe problems, or extreme problems. The responses to the five items in the EQ-5D-5L were converted into a corresponding HSUV using a conversion table. The HSUV ranges from -0.025 to 1.000, with higher scores indicating better HRQOL. The validity of EQ-5D-5L in cancer patients has been demonstrated in studies comparing it with EuroQol-5 Dimension 3-level (EQ-5D-3L) and the cancer-specific Functional Assessment of Cancer Therapy-General (FACT-G). EQ-5D-5L showed lower ceiling effects, higher test-retest reliability, and stronger convergent validity with FACT-G. It also better discriminated between cancer stages and performance statuses, supporting its use for HRQOL assessment in oncology research [22].

Body composition

Body composition was measured using a portable body composition analyzer (InBody 470, InBody Japan) that employed bioelectrical impedance analysis. Skeletal Muscle mass, appendicular skeletal muscle mass index (SMI) [23], body fat mass, body fat percentage, fat-free mass, basal metabolic rate, and waist circumference obtained from measurements were used for analysis.

Grip strength

A digital hand dynamometer (TKK 5401; Takei Scientific Instruments Co., Ltd., Niigata, Japan) was used to measure grip strength, in accordance with the guidelines of the New Physical Fitness Test Implementation Manual of the Ministry of Education, Culture, Sports, Science and Technology [24]. The grip width was adjusted such that the second joint of the index finger formed a right angle, and the measurement was conducted in a standing position with both arms hanging naturally. Each hand was measured twice, and the average of the maximum values from both hands was used as the representative value.

Gait speed

Gait speed was calculated as the time taken to walk a 10-meter distance at a usual pace [25]. Participants were instructed to "walk normally." To account for acceleration at the start and deceleration at the end of the walk, a 3-meter buffer zone was established before and after the 10-meter measurement section. Although assistive devices were permitted during the assessment, all participants completed the test without using any walking aids.

Gait independence

Gait independence was assessed using the Functional Ambulation Categories (FAC) [26], a publicly available scale. It classifies walking ability on a six-point scale ranging from 0 to 5, based on the required walking aid and degree of physical assistance. A score of 0 indicated that walking is not possible or that assistance from two or more people is required. A score of 1 indicated that walking was possible with the assistance of one person who provided continuous, close contact support. A score of 2 indicated that walking with the assistance of one person is required, but only to maintain balance, either constantly or occasionally. A score of 3 indicated that the patient could walk independently but required supervision. A score of 4 indicated independence on flat ground, and a score of 5 indicated the ability to walk independently in any environment, including on slopes.

Standing balance

Standing balance was assessed using the Standing Test for Imbalance and Disequilibrium (SIDE) and open-eye one-leg standing time. The SIDE [27,28] is a six-level scale (levels 0, 1, 2a, 2b, 3, and 4) that evaluates the ability to maintain static standing balance. It distinguishes between postures such as feet-apart, feet-together, tandem standing, and one-leg standing. In this study, the levels were recoded from 0 to 5 in ascending order and used for analysis. The SIDE is a publicly available clinical assessment tool and does not require permission for use in research settings. The open-eye, one leg standing time [24] was measured with both hands placed on each side of the body. The time was recorded using a digital stopwatch from when the foot was lifted off the ground until it touched the floor. Each side was measured twice, and the longest duration was used as the representative value. The maximum measurement time was set at 120 s.

Cognitive function

Cognitive function was assessed using the Mini-Cog [29]. The Mini-Cog is a screening test that combines three-word immediate recall, delayed recall, and a clock drawing test. Each correctly recalled word in the delayed recall was scored 1 point, and a correctly drawn clock was scored 2 points, for a total score of 5 points. A score of less than 3 indicated suspicion of dementia. To ensure the reliability of HRQOL assessments, patients with a Mini-Cog score below 3 were excluded. The Mini-Cog was selected for its ease of administration and validated accuracy, which is comparable to the Mini-Mental State Examination [29]. Permission to use the Mini-Cog for research purposes was obtained from the copyright holder.

Statistical analysis

The sample size was calculated using G*Power software (version 3.1.9.7, Germany) [30]. Based on a bivariate model with 80% power, a significance level of 5% (two-tailed), and an effect size of 0.5, a minimum sample size of 26 participants was required for correlation analysis. Data were summarized as means (standard deviations). The relationships between HSUV obtained from the EQ-5D-5L responses and each evaluation item (age, height, weight, BMI, blood test results (albumin (Alb) and hemoglobin (Hb) levels), prior chemotherapy rounds, steroid dose, indices of body composition, physical function, and cognitive function) were examined using Pearson’s and Spearman’s rank correlation coefficients, depending on normality. Multiple partial correlation analyses were also conducted. In Model 1, age and sex were controlled as covariates; in Model 2, Hb concentration was added to Model 1; and in Model 3, steroid dose was added to Model 2.

Furthermore, to identify the independent predictors of HSUV, a multiple linear regression analysis was performed. Variables that were significant in the partial correlation analysis or deemed clinically important were entered into the model as independent variables, with HSUV as the dependent variable. The model was checked for multicollinearity using the variance inflation factor (VIF).

Statistical analyses were performed using SPSS Statistics version 29.0 for Windows (IBM Corp., Armonk, NY, USA), with the significance level set at 5%. Furthermore, a post hoc power analysis was conducted using G*Power software [30] to verify the adequacy of the sample size. The effect size was set to 0.5, the α error to 0.05, and the sample size to 62, based on the inclusion criteria of this study.

## Results

**Figure 1:**
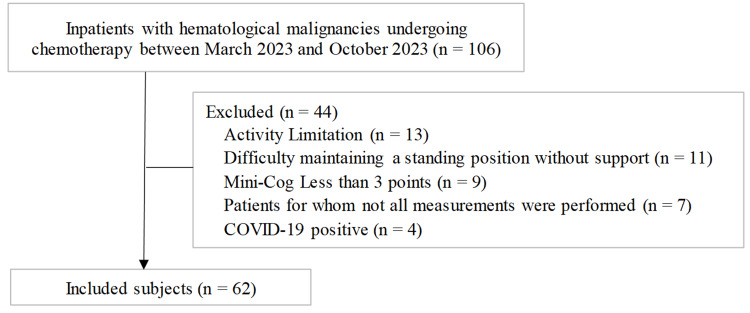
Participant recruitment

In this cross-sectional study, 106 patients newly diagnosed with hematologic malignancies were hospitalized for chemotherapy. Of these, 44 patients were excluded for the following reasons: restricted physical activity (n = 13), inability to maintain a standing position without support (n = 11), Mini-Cog scores < 3 (n = 9), incomplete assessments (n = 7), and COVID-19 positivity (n = 4). Accordingly, 62 patients met the inclusion criteria and were included in the final analysis (Figure 1).

The demographic and clinical characteristics of the patients are presented in Table 1. The mean age was 71.7 ± 13.3 years, and 48.4% of the patients were female. According to the BMI classification, 67.7% of patients were of normal weight, while 16.1% were underweight. The most common diagnosis was lymphoma (51.6%), of which aggressive lymphoma was the most frequent subtype.

**Table 1:** Demographic and clinical characteristics of the patients (n=62) Data are presented as mean ± SD (standard deviation) or median IQR (interquartile range); n is shown where applicable. BMI classification: underweight (<18.5 kg/m²), normal weight (18.5–24.9 kg/m²), overweight (25.0–29.9 kg/m²), and obese (>=30.0 kg/m²). BMI: Body Mass Index. Alb: Albumin level. Hb: Hemoglobin concentration.

Characteristics	
Age, years	71.7 ± 13.3
Sex, n (%)	
Female	30 (48.4%)
Men	32 (51.6%)
Height, cm	158.5 ± 10.2
Weight, kg	54.4 ± 9.8
BMI, kg/m²	21.6 ± 2.9
BMI classification, n (%)	
Underweight	10 (16.1%)
Normal weight	42 (67.7%)
Overweight	10 (16.1%)
Obese	0
Waist circumference, cm	74.3 ± 8.2
Blood test results	
Alb, g/dL	3.8 ± 0.4
Hb, g/dL	10.5 ± 2.2
Diagnosis, n (%)	
Lymphoma	32 (51.6%)
Aggressive lymphoma	30 (48.4%)
Indolent lymphoma	2 (3.2%)
Myelodysplastic syndrome	10 (16.1%)
Acute myeloid leukemia	9 (14.5%)
Acute lymphoblastic leukemia	5 (8.1%)
Multiple myeloma	5 (8.1%)
Chronic myeloid leukemia	1 (1.6%)
Prior chemotherapy rounds	1.0 [0.0–4.0]
Categorized, n (%)	
None	28 (45.2%)
One	8 (12.9%)
Two or more	26 (41.9%)
Steroid dosage, mg/day	0.0 [0.0–0.0]

The results of the body composition, physical function, and HRQOL assessments are shown in Table 2. The mean Health State Utility Value (HSUV), derived from the EQ-5D-5L, was 0.799 ± 0.140.

**Table 2:** Body composition, physical function, and HRQOL measures (n = 62) Data are presented as mean ± SD (standard deviation) or median [IQR (interquartile range)]. SIDE is scored from 0 to 5, with levels 0, 1, 2a, 3, and 4 converted in ascending order. SMI: Skeletal Muscle mass Index. FAC: Functional Ambulation Categories. SIDE: Standing Test for Imbalance and Disequilibrium. EQ-5D-5L: EuroQol 5-Dimension 5-Level. HSUV: Health State Utility Value.

Assessment measures	
Skeletal muscle mass, kg	21.2 ± 4.5
SMI, kg/m²	6.2 ± 1.0
Body fat mass, kg	13.3 ± 4.9
Body fat percentage, %	24.7 ± 7.0
Lean body mass, kg	40.0 ± 7.5
Basal metabolic rate, kcal	1255.0 ± 198.9
Mini-Cog, score	5.0 [5.0–5.0]
Grip strength, kg	23.0 ± 8.6
Gait speed, m/sec	0.98 ± 0.23
FAC	5.0 [4.0–5.0]
SIDE	4.0 [3.0–5.0]
One leg standing time, sec	9.5 [4.0–30.0]
EQ-5D-5L, HSUV	0.799 ± 0.140

Table 3 shows the correlation between HSUV and the results of each assessment. In the simple bivariate correlation analysis, HSUV was significantly associated with various physical characteristics, including age, weight, BMI, skeletal muscle mass, SMI, grip strength, gait speed, and FAC. In the partial correlation analysis, after adjusting for potential confounders (Models 1-3), weight, BMI, body fat percentage, gait speed, and FAC maintained a significant correlation with HSUV.

**Table 3:** Correlation between HSUV and the results of each assessment (n=62) ** p<0.01 * p<0.05 aAge and sex were included as control variables bIn addition to Model 1, Hb was also included as a control variable cIn addition to Model 2, Steroid dosage was also included as a control variable. BMI: Body Mass Index, Alb: Albumin level, Hb: Hemoglobin concentration, SMI: Skeletal Muscle mass Index, FAC: Functional Ambulation Categories, SIDE: Standing Test for Imbalance and Disequilibrium, HSUV: Health State Utility Value.

Variables	Correlation coefficient (n = 62)	Partial correlation coefficient (n = 62)					
		Model 1^a^		Model 2^b^		Model 3^c^	
Age	-.267*	―		―		―	
Height	.372**	.099		.117		.153	
Weight	.455**	.304*		.289*		.300*	
BMI	.312*	.344**		.323*		.317*	
Waist circumference	.306*	.191		.150		.187	
Alb	.144	.022		-.017		-.009	
Hb	.195	.139		―		―	
Total number of chemotherapy	.012	-.133		-.140		-.152	
Steroid dosage	-.023	-.139		-.157		―	
Skeletal muscle mass	.308*	.007		-.008		-.005	
SMI	.380**	.179		.170		.162	
Body fat mass	.178	.236		.206		.223	
Body fat percentage	.078	.294*		.268*		.292*	
Lean body mass	.291*	-.011		-.019		-.017	
Basal metabolic rate	.163	-.091		-.085		-.089	
Mini-Cog	.192	.052		.058		-.024	
Grip strength	.525**	.193		.165		.172	
Gait speed	.613**	.529**		.528**		.519**	
FAC	.609**	.623**		.625**		.618**	
SIDE	.299*	.232		.219		.244	
One leg standing time	.368**	.112		.118		.126	

To identify independent predictors of HSUV, we performed a multiple linear regression analysis, selecting independent variables based on prior analyses and clinical relevance (age, sex, BMI, body fat percentage, gait speed, and FAC). BMI was chosen over weight to adjust for height and reduce multicollinearity. The results (Table 4) showed that only gait speed (β = 0.304, p = 0.047) and FAC (β = 0.411, p = 0.005) remained significant independent predictors of HSUV. In contrast, variables such as BMI and body fat percentage were not statistically significant. The model explained 50.5% of the variance in HSUV (adjusted R² = 0.505).

**Table 4:** Multiple regression analysis of factors associated with HSUV (n = 62) BMI: Body Mass Index, FAC: Functional Ambulation Categories, HSUV: Health State Utility Value.

Parameters	Standardized regression coefficient (β)	p-value
Age	0.031	0.781
Sex	–0.093	0.402
BMI	0.185	0.126
Body fat percentage	0.033	0.809
Gait speed	0.304	0.047
FAC	0.411	0.005
Adjusted R² = 0.505		

Post hoc power analysis using G*Power indicated a power (1 - β error) of 0.99, demonstrating sufficient statistical power to detect significant differences for the assessed variables.

## Discussion

Our findings demonstrate that gait ability is a key determinant of HRQOL in hospitalized patients with hematologic malignancies. Both gait speed and the FAC were identified as significant independent predictors of HRQOL. These measures reflect a patient's overall walking ability, including performance and level of independence. This underscores that maintaining mobility is crucial for preserving HRQOL in this population.

In fact, reduced mobility has been linked to declines in global QOL in older cancer patients [31], and conversely, interventions that improve walking ability can yield tangible benefits. For example, exercise trials have shown that walking programs can preserve functional status and alleviate symptoms like fatigue and distress, with one study of hospitalized leukemia patients demonstrating that even brief walking reduced fatigue intensity and improved emotional well-being [32]. The benefits of such interventions extend to other populations; for instance, a randomized trial in transplant recipients found that a structured walking regimen helped maintain functional capacity during recovery, which is expected to positively influence QOL [33]. These observations align with our results, indicating that patients who walk faster and more independently tend to enjoy better HRQOL.

Importantly, the impact of walking ability on QOL likely extends beyond just physical fitness to encompass autonomy and social participation. Higher FAC reflects greater ambulatory independence, which enables patients to perform daily activities, engage with others, and fulfill social roles. Prior studies have demonstrated a strong link between independence and life satisfaction in older adults, showing that those with impaired mobility report significantly lower life satisfaction. In contrast, the ability to get around independently allows one to remain involved in meaningful activities and society, a factor that can be even more influential on life satisfaction than basic ADL ability alone [34]. Thus, beyond preserving “physical function,” maintaining mobility means preserving independence and social participation, which are key contributors to quality of life in older people. Our finding that FAC is an independent HRQOL predictor supports this, suggesting that helping patients remain mobile and self-sufficient may bolster not only their physical health but also their sense of autonomy and well-being.

Another notable result is that nutritional or body composition measures (e.g., BMI and body fat percentage) did not retain significance in the multivariate analysis once gait speed and FAC were accounted for. This does not mean that nutritional status is unimportant - indeed, our univariate correlations showed BMI, weight, and fat were positively associated with HRQOL scores, consistent with extensive literature linking poor nutrition to worse QOL in cancer patients.

Many studies have documented that malnutrition and weight loss accompany diminished QOL. For example, although the study focused on hematopoietic transplant recipients, one trial observed that severely malnourished patients had lower QOL scores compared to their well-nourished counterparts [35]. Likewise, in a study of oncology outpatients, those at risk of malnutrition reported significantly poorer QOL than well-nourished patients [36]. A systematic review of epidemiologic studies confirms a strong association between better nutritional status and better QOL outcomes in cancer patients [37].

Given this evidence, one might expect anthropometric indicators to influence HRQOL. However, our multivariable model suggests that functional capacity outweighs these static measures in our study population of 62 hospitalized patients with hematologic malignancies. One interpretation is that, beyond a baseline level of nutrition, what ultimately matters more for quality of life is how well patients can function in daily life. In other words, static indicators such as muscle mass, body weight, and body fat percentage may not in themselves translate to better QOL unless accompanied by the functional ability to engage in meaningful activities.

This perspective is supported by prior research highlighting that functional status can be more critical than body composition for QOL in advanced cancer. For instance, a large study of incurable cancer patients demonstrated this contrast clearly: while poor Eastern Cooperative Oncology Group Performance Status (ECOG PS) 3-4 was one of the most powerful independent predictors of low QOL, measures of muscle mass were not [38]. In other words, being physically frail or functionally impaired had a more direct impact on QOL than simply having low muscle or body fat. Our results echo this pattern: gait speed and walking independence explained a substantial portion of the variance in HSUV (adjusted R² ~0.50), rendering BMI and adiposity non-significant in the model. The majority of patients in our sample were of normal body weight. For these hospitalized patients with hematologic malignancies, it appears that variability in HRQOL is driven more by differences in functional mobility than by differences in body composition per se. This aligns with the geriatric oncology concept that functional status is more crucial to outcomes than nutritional status alone. A key study supporting this found that in older adults undergoing cancer treatment, baseline mobility impairment and depressive symptoms predicted worse functional and QOL trajectories, leading the authors to conclude that preserving functional abilities offers the greatest payoff for patients’ day-to-day QOL [31].

It is worth noting that the relationship between body composition and QOL in cancer survivors can be complex. For instance, in patients with breast cancer, changes in body fat or lean mass during treatment were not significantly associated with changes in quality of life, suggesting that body composition may not always be a key determinant of QOL [39]. In contrast, a prospective study of colorectal cancer survivors found that post-treatment increases in both adipose tissue and muscle function were longitudinally associated with better HRQOL and less fatigue [40]. These findings suggest that, for survivors recovering from treatment, restoring body tissues-rather than simply minimizing adiposity-may be beneficial for improving their QOL.

In our cross-sectional study of older adults (mean age >70), it is possible that most patients were not severely malnourished (only 16% were underweight) and that physical function was the more discriminating factor for HRQOL. We also did not directly measure ≥5% weight loss over the past six months, which might affect QOL if present. Nonetheless, the dominance of gait speed and FAC in our regression implies that improving a patient’s functional status could yield more immediate QOL benefits than modest changes in weight or body fat. From a clinical standpoint, this finding reinforces the importance of interventions aimed at maintaining or enhancing mobility in hospitalized patients with hematologic malignancies. Importantly, gait speed and FAC are not only clinical indicators but also have the potential to improve through individualized mobility exercises during hospitalization, which may help support or enhance HRQOL.

Overall, our study suggests that “moving well” is integral to “feeling well” for hospitalized patients with hematologic malignancies. While managing disease and nutritional status remains essential, equal emphasis should be placed on rehabilitative and supportive strategies that help patients remain active. Simple gait assessments can serve as practical tools to identify individuals at risk of QOL deterioration. Furthermore, targeted interventions-such as physical therapy, ambulatory training, or structured exercise programs-may not only improve physical function but also enhance patients’ independence and social engagement, thereby enriching their overall QOL. Future research should investigate whether improvements in gait speed or FAC through rehabilitation can directly translate into measurable gains in HRQOL in this population. Our findings, supported by a high post-hoc power (0.99) and consistent with prior evidence, provide a strong rationale for incorporating mobility-focused interventions as a core component of holistic cancer care for hospitalized patients with hematologic malignancies. By prioritizing functional ability alongside traditional oncologic and nutritional care, we may more effectively support the overall well-being of older patients throughout their cancer journey.

This study has several limitations. First, its cross-sectional design precludes causal inference as to whether improved walking ability directly contributes to better HRQOL in hospitalized patients with hematologic malignancies. Second, cancer-specific performance scales such as the ECOG PS were not assessed in this study. This omission may limit direct comparisons with other cancer-related studies that commonly utilize such scales. However, our study incorporated detailed assessments of physical function, including grip strength, gait speed, gait independence, and standing balance, alongside body composition parameters, allowing for a comprehensive evaluation of patients’ physical status. Nevertheless, we did not assess patients’ baseline physical activity levels or sedentary behavior prior to hospitalization, which may have influenced HRQOL independently of their measured physical performance. Third, patients were excluded if they exhibited cognitive impairment based on a Mini-Cog score below 3. Additionally, those who were unable to stand independently were also excluded due to the physical assessment requirements. While these criteria ensured the reliability of HRQOL responses and the feasibility of physical measurements, they may have introduced selection bias by excluding more vulnerable individuals, such as those with severe frailty or impaired mobility. This could lead to an underestimation of the true association between impaired physical function and HRQOL in the broader hospitalized population. Fourth, this study included patients with both acute and chronic hematologic malignancies undergoing a range of chemotherapy protocols. The heterogeneity in treatment types, intensities, and symptom burdens may have influenced the observed associations between physical function and HRQOL. Fifth, this study lacked a follow-up assessment after chemotherapy. Consequently, we were unable to evaluate how treatment-related changes in physical function or body composition may have influenced HRQOL over time. Longitudinal data will be essential.

Despite these limitations, this study aimed to provide an inclusive assessment of the relationships between physical characteristics and HRQOL. Future research should incorporate stratified analyses based on disease subtypes and treatment regimens to enhance the generalizability of findings. Additionally, prospective studies should evaluate the effectiveness of physical rehabilitation programs designed to maintain or improve walking ability, in order to preserve and enhance HRQOL in this patient population.

## Conclusions

Preserving and enhancing functional mobility, particularly gait speed and gait independence, should be prioritized in supportive care for hospitalized patients with hematologic malignancies. While nutritional support remains important, functional mobility appears to have a greater influence on HRQOL. Promoting physical rehabilitation may help maintain patients’ autonomy and HRQOL during treatment.

## References

[1] Ribera JM, García-Calduch O, Ribera J (2022). Ponatinib, chemotherapy, and transplant in adults with Philadelphia chromosome-positive acute lymphoblastic leukemia. Blood Adv.

[2] Röllig C (2023). Improving long-term outcomes with intensive induction chemotherapy for patients with AML. Hematology Am Soc Hematol Educ Program.

[3] Zhang N, Wu J, Wang Q (2023). Global burden of hematologic malignancies and evolution patterns over the past 30 years. Blood Cancer J.

[4] Fukushima T, Nakano J, Ishii S, Natsuzako A, Hirase T, Sakamoto J, Okita M (2019). Characteristics of muscle function and the effect of cachexia in patients with haematological malignancy. Eur J Cancer Care (Engl).

[5] Xiong J, Chen K, Huang W, Huang M, Cao F, Wang Y, Chen Q (2023). Prevalence and effect on survival of pre-treatment sarcopenia in patients with hematological malignancies: a meta-analysis. Front Oncol.

[6] Großek A, Großek K, Bloch W (2023). Safety and feasibility of exercise interventions in patients with hematological cancer undergoing chemotherapy: a systematic review. Support Care Cancer.

[7] Kazuma K (2006). Measurement of health-related quality of life (HR-QOL) in cancer patients. Fam Tumor.

[8] Shimozuma K, Noto S (2023). Manual for QOL evaluation useful in clinical and research settings. Igaku-Shoin, Tokyo.

[9] Kingsley C, Patel S (2017). Patient-reported outcome measures and patient-reported experience measures. BJA Educ.

[10] Fukuhara T (2002). QOL evaluation and epidemiology for clinical practice. J Jpn Lumbar Spine Soc.

[11] Shimozuma K (2015). History and prospects of QOL evaluation research. Jpn J Behav Med.

[12] Allart-Vorelli P, Porro B, Baguet F, Michel A, Cousson-Gélie F (2015). Haematological cancer and quality of life: a systematic literature review. Blood Cancer J.

[13] La Nasa G, Caocci G, Morelli E (2020). Health related quality of life in patients with onco-hematological diseases. Clin Pract Epidemiol Ment Health.

[14] Konstantinidis TI, Tsatsou I, Tsagkaraki E, Chasouraki E, Saridi M, Adamakidou T, Govina O (2024). Quality of life and symptoms of hospitalized hematological cancer patients. Curr Oncol.

[15] Fukushima T, Nakano J, Ishii S (2019). Influence of hemoglobin level on muscle and physical functions, activities of daily living, and quality of life in patients with hematological malignancies. Integr Cancer Ther.

[16] Fukushima T, Nakano J, Ishii S (2020). Factors associated with muscle function in patients with hematologic malignancies undergoing chemotherapy. Support Care Cancer.

[17] Fukushima T, Nakano J, Ishii S, Natsuzako A, Sakamoto J, Okita M (2018). Low-intensity exercise therapy with high frequency improves physical function and mental and physical symptoms in patients with haematological malignancies undergoing chemotherapy. Eur J Cancer Care (Engl).

[18] McGowan K (2016). Physical exercise and cancer-related fatigue in hospitalized patients: role of the clinical nurse leader in implementation of interventions. Clin J Oncol Nurs.

[19] Borsati A, Murri A, Natalucci V (2025). The effect of exercise-based interventions on health-related quality of life of patients with hematological malignancies: a systematic review and meta-analysis. Healthcare (Basel).

[20] Herdman M, Gudex C, Lloyd A (2011). Development and preliminary testing of the new five-level version of EQ-5D (EQ-5D-5L). Qual Life Res.

[21] Ikeda S, Shiroiwa T, Igarashi A, Noto S, Fukuda T, Saito S, Shimozuma K (2015). Developing a Japanese version of the EQ-5D-5L value set. Jpn J Health Med Sci.

[22] Zeng X, Sui M, Liu B (2021). Measurement properties of the EQ-5D-5L and EQ-5D-3L in six commonly diagnosed cancers. Patient.

[23] Arai H, Ikegawa N, Nozoe M, Kamiya K, Matsumoto S (2022). Association between skeletal muscle mass index and convalescent rehabilitation ward achievement index in older patients. Prog Rehabil Med.

[24] (2024). Ministry of Education, Culture, Sports, Science, and Technology New physical fitness test implementation guidelines (for ages 65-79). Sports, Science, and Technology New physical fitness test implementation guidelines (for.

[25] Ando M, Maruyama H, Kosaka K (1995). Effect of different walking speeds on comfortable walking. Jpn J Phys Ther.

[26] Mehrholz J, Wagner K, Rutte K, Meissner D, Pohl M (2007). Predictive validity and responsiveness of the functional ambulation category in hemiparetic patients after stroke. Arch Phys Med Rehabil.

[27] Kondo I, Hosokawa K, Iwata M (2004). Examination of the inter-rater reliability of the standing test for imbalance and disequilibrium (SIDE). Jpn J Rehabil Med.

[28] Teranishi T, Kondo I, Sonoda S (2010). A discriminative measure for static postural control ability to prevent in-hospital falls: Reliability and validity of the Standing Test for Imbalance and Disequilibrium (SIDE). Jpn J Compr Rehabil Sci.

[29] Borson S, Scanlan JM, Chen P, Ganguli M (2003). The Mini-Cog as a screen for dementia: validation in a population-based sample. J Am Geriatr Soc.

[30] Faul F, Erdfelder E, Lang AG, Buchner A (2007). G*Power 3: a flexible statistical power analysis program for the social, behavioral, and biomedical sciences. Behav Res Methods.

[31] Kirkhus L, Harneshaug M, Šaltytė Benth J (2019). Modifiable factors affecting older patients' quality of life and physical function during cancer treatment. J Geriatr Oncol.

[32] Chang PH, Lai YH, Shun SC (2008). Effects of a walking intervention on fatigue-related experiences of hospitalized acute myelogenous leukemia patients undergoing chemotherapy: a randomized controlled trial. J Pain Symptom Manage.

[33] DeFor TE, Burns LJ, Gold EM, Weisdorf DJ (2007). A randomized trial of the effect of a walking regimen on the functional status of 100 adult allogeneic donor hematopoietic cell transplant patients. Biol Blood Marrow Transplant.

[34] Misu Y, Hayashi S, Iwai N, Yamamoto T (2022). Factors affecting the life satisfaction of older people with care needs who live at home. Geriatrics (Basel).

[35] Cioce M, Botti S, Lohmeyer FM (2022). Nutritional status and quality of life in adults undergoing allogeneic hematopoietic stem cell transplantation. Int J Hematol.

[36] Sonneborn-Papakostopoulos M, Dubois C, Mathies V, Heß M, Erickson N, Ernst T, Huebner J (2021). Quality of life, symptoms and dietary habits in oncology outpatients with malnutrition: a cross-sectional study. Med Oncol.

[37] Lis CG, Gupta D, Lammersfeld CA, Markman M, Vashi PG (2012). Role of nutritional status in predicting quality of life outcomes in cancer--a systematic review of the epidemiological literature. Nutr J.

[38] Daly LE, Dolan RD, Power DG (2020). Determinants of quality of life in patients with incurable cancer. Cancer.

[39] Porciúncula Frenzel A, Aberici Pastore C, González MC (2013). The influence of body composition on quality of life of patients with breast cancer. Nutr Hosp.

[40] Kenkhuis MF, van Roekel EH, Koole JL (2021). Increases in adipose tissue and muscle function are longitudinally associated with better quality of life in colorectal cancer survivors. Sci Rep.

